# Germs of Disease

**Published:** 1882-11

**Authors:** 


### GERMS OF DISEASE.

### SPREAD OF CONTAGION—USELESSNESS OF QUARANTINE.

IN Chinese towns, where the streets reek with the vilest odors, the contagious fevers are almost unknown. The free contact of air with the filth prevents the formation, probably by complete oxidation, of the albuminoid element of morbific matter. Epidemic diseases, again, are often classed as contagious without sufficient reason. Influenza, for instance, is common in London during the prevalence of the east wind, and member after member of a household is attacked by it, one being said to catch it from the other. But the true explanation, probably, is that living under like conditions, they are similarly influenced by a common cause. It may be very well doubted whether the spread of cholera may not be mainly accounted for in like manner. Of this terrible disease, the contagion is, undoubtedly, often conveyed in water; but the mode of origin and rapidity of development of cholera epidemics point to the existence of agencies of a speedier and more ubiquitous operation. The epidemic that visited Japan in 1877 could not be traced to any foreign source. It was asserted that it was imported from China, but no definite proof of the assertion was produced. The spread of the epidemic was extremely rapid, and its incidence singularly sporadic, phenomena hardly explicable by reference to two or three points of origin, or otherwise than on the theory of the presence of a widely prevalent cause. In Yokohama, among the Chinese, some 1,500 in number, living under exactly the same conditions as the Japanese, but better fed and of a stronger physique, not a single case occurred. The villages along the high road between Tokio and Kiyoto were not all attacked; here and there one suffered, the rest escaped; a proof that the poison was only active where fitting conditions were present. Of scarlatina and small-pox, no doubt, the contagion is much more virulent. It may be conveyed in a letter, left in an old stocking, hang for months about a house, reside in the breath, or exhale from the skin.

If compulsory isolation should be justifiable at all, it would be in respect to these two diseases. But, in reality, the same reasoning applies to these as to other contagious or epidemic diseases, but with somewhat less force. The poison of small-pox and scarlatina, like that of typhoid, is sometimes, no doubt, of spontaneous origin, much more frequently so, in all probability, than is commonly suspected. We are surrounded by morbific germs of all kinds, unstable albuminoids that only need the aid of certain well-known conditions to develop into deadly poisons. We can no more stamp out these germs than we can stamp out mosquitoes. In all medical books we read of "predisposing causes"—bad ventilation, crowded habitations, ill-drained soils, low, marshy situations, insufficient or improper food, overwork, intemperate habits and the like. These so called "predisposing causes" are more probably necessary conditions precedent of the development of disease. Men in good health and of "sanitary" habits may and do expose themselves with impunity to the most concentrated forms of contagion. Nobody proposes to isolate doctors. Hence, as the germs of infectious disease cannot be reached, while the conditions of their development and, to a greater or less extent, of their birth, may be removed by ordinary sanitary measures, it would appear preferable to direct our efforts mainly toward the perfection of these. The black death of the Middle Ages and the plague of later times have disappeared from all civilized countries, simply through the amelioration of the hygienic conditions of life. Much still remains to be done, but we may reasonably hope that in like mannor the continued progress of sanitary sience will result in the extinction of even such diseases as small pox and scarlatina, without recourse to inquisitorial and violent legislative measures. Quarantine has been condemned over and over again as a means of preventing the importation of disease. In no single case has it kept out a contagious or epidemic disorder. It inflicts enormous loss, inconvenience, and even suffering upon perfectly healthy and hygienically innocent persons.—*London Spectator.*

